# Soft Tissue Filler Therapy and Informed Consent: A Canadian Review

**DOI:** 10.1177/12034754211032542

**Published:** 2021-07-26

**Authors:** John P. Arlette, Andrea L. Froese, Jaspreet K. Singh

**Affiliations:** 1 70401 Department of Surgery, Cumming School of Medicine, University of Calgary, Alberta, Canada; 2 113536 Bennett Jones LLP, Calgary, Alberta, Canada

**Keywords:** informed consent, aesthetics, Soft Tissue Filler (STF), risk factors, litigation, Canadian Medical Protective Association (CMPA), patient’s satisfaction

## Abstract

Soft Tissue Filler (STF) Therapy for cosmetic facial rejuvenation is associated with known complications. The manifestation of these known complications can lead to patients commencing civil litigation actions or making complaints to provincial regulatory authorities and alleging that the practitioner failed to obtain the patient’s informed consent to the therapy. Data provided by the Canadian Medical Protective Association (CMPA) on medical-legal cases arising from the provision of STF therapy between 2005 and 2019 are presented. Select reported case law decisions from Canadian courts and regulatory bodies addressing the concept of informed consent are reviewed. Insights about the risk factors pertaining to the process of obtaining informed consent for STF therapy are presented to increase an understanding of the elements of communication and documentation needed to ensure patients are aware of the consequences of this treatment.

## Introduction

The use of Soft Tissue Fillers (STFs) to enhance facial features in a non-invasive way has expanded patients’ options to obtain cosmetic results in a clinic setting with minimal downtime. There are several known complications that can occur as a direct result of STF injections. The most significant adverse events can occur at the time of injection, such as vascular occlusions and blindness.^1,2^ Other adverse events include those that develop soon after the treatment, such as infection, or those that occur several months later, with the appearance of inflammatory nodular reactions.

The incidence of complications from STF is low. However, the increased utilization of STF for cosmetic services is associated with the heightened frequency of the known consequences of treatments. In addition to the direct adverse event, there is a possibility that patients may experience unsatisfactory results or unmet expectations. Physicians who inject STF for cosmetic corrections may be involved in medical-legal disputes or receive complaints to regulatory authorities from patients who perceive that they have experienced harm as a direct result of their treatment

We review all of the data provided by the Canadian Medical Protective Association (CMPA) on medical-legal cases arising from the provision of dermal filler therapy between 2005 and 2019 (CMPA STF Data). We also reviewed reported medical-legal cases related to STF therapy in Canada between 2005 and 2019 (STF Decisions). From this information, we identify factors presented in the CMPA STF Data and STF Decisions relating to informed consent to highlight those issues for practitioners of STF therapy.

## Materials and Methods

### Study Context

The CMPA has more than 100,000 physician members, representing over 95% of Canadian doctors.^3^ The CMPA maintains a national database that includes information on advice calls to the Association, civil legal actions, and complaints to hospitals and provincial regulatory authorities known as Colleges.

### Case Identification

In response to a request for information, CMPA medical analysts reviewed records, legal documents, peer expert opinions, regulatory authority and hospital decisions to provide data to respond to the question “What are the medical-legal risks associated with administration of dermal fillers?”

### Data Acquisition

The CMPA analyzed its database for medical-legal cases from all types of member work and specialty over the interval January 2005 and July 2019 for closed civil legal cases, College complaints and Hospital cases in all settings for the provision of dermal filler therapy, resulting in the CMPA STF Data. Publicly available civil and regulatory decisions in Canada involving STF therapy between 2005 and 2019 were identified using the following search terms: Dermal Fillers, Soft Tissue Fillers, Fillers, Autologous Fat, Hyaluronic Acid Gel, Hyaluronic acid Filler, HA filler, Juvederm, Voluma, Volift, Volbella, Restylane, Teosyl, Radiesse, Sculptra, Poly L Lactic Acid, Dermadeep, DermaLive, Artecoll, PolyMethalMethacrolate, Silicone, Facial Volumizer, Lip augmentation, Lip Filler. These terms were searched in the following legal databases as individual terms and as a large search string: CanLII, Westlaw, Quicklaw and MedLaw. These searches resulted in the STF Decisions.

## Results of CMPA STF Data

In the time period evaluated, there were 85,191 medical-legal cases. 41,258 cases were closed with final determinations, comprehensive medical-legal information and peer expert criticism for in-depth analysis. There were 90 CMPA medical-legal cases involving 92 physicians related to dermal fillers. Civil medical-legal actions represented 60% (54/90) of the cases, and College complaints 40% (36/90). The proportion of physician unfavourable outcomes in cases involving dermal fillers was 54% (49/90) versus a historical rate of 47% (19,496/41,258) for the 14-year interval 2005-2019 (Table 1).

**Table 1:** Physician Work Type of the 92 Medical Practitioners Involved in the 90 cases.

Medical specialty	Number (%)
Family physician	38 (41)
Plastic surgeon	23 (25)
Dermatologist	12 (13)
Otolaryngology	9 (10)
Surgical services	5 (5)
(Emergency medicine, ophthalmology, anesthesia accounted for individual physicians)	N/A

Fillers containing hyaluronic acid (HA) were associated with a higher incidence of localized swelling or edema compared to non-HA fillers. Rare complications such as arterial occlusion and retinal vascular occlusion were not associated with HA fillers (Table 2).

**Table 2:** Types of Dermal Fillers Involved in Cases, More Than One Type of Filler Was Injected in Some Instances.

Dermal filler type	Number of cases (%)
Hyaluronic acid	57 (59)
Acrylic acid, calcium hydroxy appetite, fat grafts, silicone, poly-L-lactic acid	17 (18)
Unknown	13 (14)
Collagen	9 (9)

The issue of inadequate consent in the CMPA STF Data related to three areas. First, allegations about inadequate consent concerned the risks, limitations, side effects, type and costs of STF, as well as communicating post-treatment aesthetic expectations. The second issue relates to the procedure being delegated to another health care providers and appropriate information about the person performing the controlled act. The final issue relating to inadequate consent identified related to ensuring the patient has a clear understanding of the procedure.

Healthcare-related harm, involving a negative effect on the patient’s health or quality of life, occurred in 65% (56/90) of the 90 patients. “Healthcare-associated harm” is defined by the CMPA Data as follows: Harm arising from, or associated with, plan or actions taken during the provision of healthcare, rather than an underlying disease or injury. Mild harm is defined as where the patient is symptomatic with mild symptoms, minimal or no intervention is required, and causing minimal impact on physical, mental, or social function. Avoidable harm from the patient’s clinical care occurred in 68% (38/56). No harm was assessed in 29% (11/38) where it was determined that a harmful incident had occurred, but the patient remained asymptomatic. No harm definition by CMPA Data. “No harm” is defined by the CMPA Data as being where no symptoms were detected and no treatment required.

Healthcare-related harm occurred in the face of well-managed care in 32% (18/56), where it was determined that the harm was an inherent risk of the medication or treatment (Table 3).

**Table 3:** Most Common Types of Patient Complaints (Some Cases Involved More Than One Complication).

Types of patient complaints	Number
Disorders of the skin & subcutaneous tissue (granuloma at the injection site, scar formation)	22
Localized edema swelling or lump formation	18
Infection at the injection site	6
Rare complications (tissue necrosis, arterial occlusion, blindness)	5

**Figure 1:**
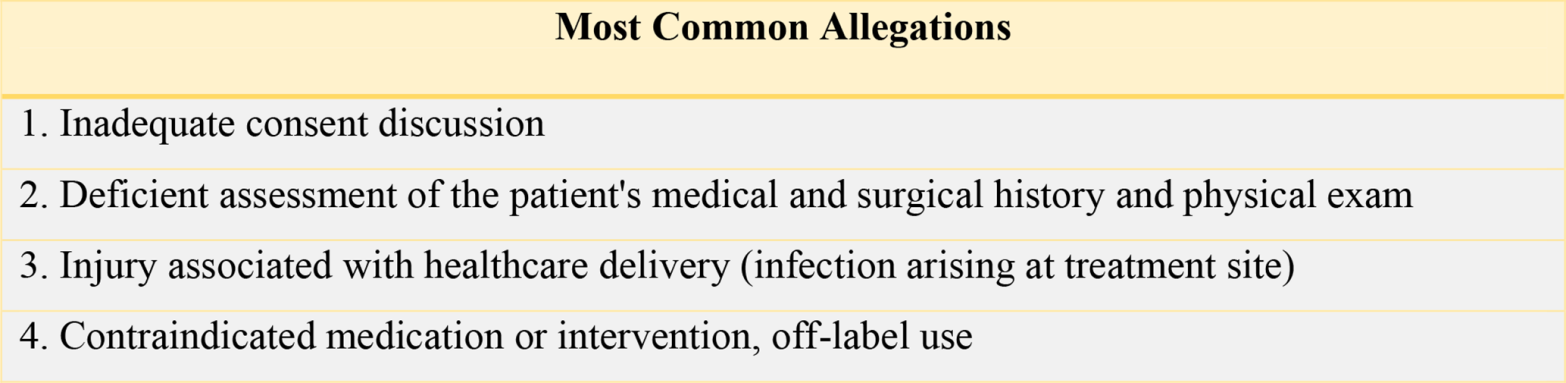
Allegations related to patient care reflect the perception of the problems that occurred during care.

**Figure 2:**
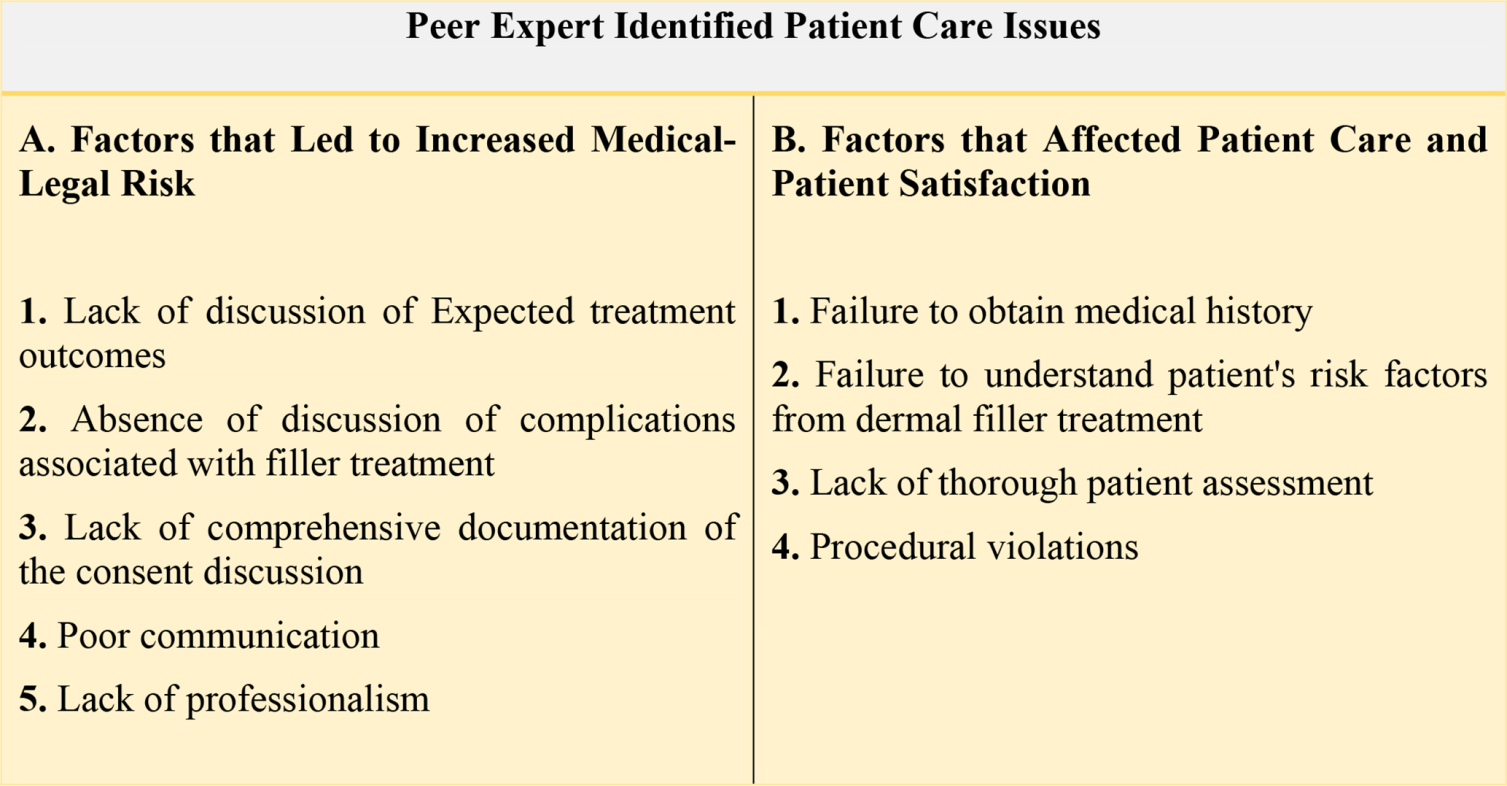
Summary of peer expert identified patient care issue.

Peer expert criticism of care was levelled in 52% (47/90) of cases; these were analyzed and categorized as contributing components of provider, team or system factors. Provider factors like physician clinical decision-making, health, conduct, boundary issues, and procedural violations were identified in 62% (29/47). In this group of provider factors, 62% (18/29) were related to clinical decision making, 38% (11/29) to health conduct and boundary issues, and 31% (9/29) to procedural variations. Team factors were identified in 57% (27/47) cases where there was a breakdown in communication with the patient or inadequate documentation (Figures 1 and 2).

## Discussion

From the CMPA STF Data, there were 90 closed medical-legal cases that involved dermal fillers in Canada during the 14-year period studied (Figure 1). Although 62% of patients experienced healthcare-related harm, the level of harm in the majority of cases was classified as mild or no harm. Even though the number of STF treatments carried out in Canada during that same time is unknown, this number of medical-legal cases represents a fraction of the patients who received STF therapy in the cosmetic context. The data show the medical specialty of the practitioners involved in the cases came from a wide range of disciplines (Table 1). The first three groups - family physicians, plastic surgeons and dermatologists—formed 79% of the physicians responsible for patient care (Table 1).

The type of STF used in the treatment resulting in complications includes examples from almost the entire range of products used in STF treatments. Hyaluronic acid was a product in 59% of medical-legal cases, likely reflecting its use frequency (Table 2). None of the incidences of vascular occlusion involved the use of this product. It is not known whether this is related to the fact that Hyaluronic acid gel fillers can be in part dissolved by the use of hyaluronidase. However, while all product groups used in injectable therapy are represented, no conclusion can be drawn as to the frequency of complications inherent with each particular type of dermal filler.

In our review of legal decisions, the search terms used for the publicly available civil and regulatory body Canadian decisions between 2005 and 2019 produced a total of 14 STF Decisions which addressed a variety of medical-legal issues associated with STF therapy. We provide a brief summary of each of the STF Decisions’ key aspects in Supplemental Table S1.

As illustrated in Table A, the STF Decisions included civil, criminal, and regulatory decisions, with approximately 45% civil cases. It is important to understand when considering that there are only 14 reported decisions that complaints made to a regulatory body in Canada may only become public if they proceed to a hearing. Accordingly, the number of STF Decisions in the regulatory sphere cannot be assumed to represent the totality of complaints made to the regulatory body involving STF therapy. Given the accessibility of the regulatory process to patients as compared to civil litigation and the type of complications associated with STF therapy, it is reasonable to assume that it is in the regulatory venue that physicians will most often encounter allegations concerning lack of informed consent for STF therapy.

### Informed Consent and General Legal Principles

The development of the law on informed consent demonstrates an evolving understanding of the physician-patient relationship as one that requires communication and patient control over treatment and care. A central concept arising from a patient’s right to self-determination is the physician’s obligation to obtain informed consent from their patients by providing them with disclosure of the nature, risks, and benefits of options for treatment.

The modern concept of informed consent entered the medico-legal landscape in the 1980s following a series of decisions from the Supreme Court of Canada..^4,5^ In those decisions, the Supreme Court of Canada discussed the types of risks that must be disclosed by a physician, namely any “material risks and any special or unusual risk” (*Ref 6, page 193*).^4^

A material, special, or unusual risk has been interpreted to mean anything that a reasonable person in the patient’s position would want to know in deciding whether or not to undergo the proposed therapy. Material, special or unusual risks include those that are significant and pose a real threat to the patient’s life, health, and comfort (*Ref 7, page 884-885, Ref 9 page 166-174*).^4,5^ The severity of the potential adverse event and the likelihood of it occurring factor into the assessment of whether a risk is material, special, or unusual; for example, a minimal risk of death or serious injury may be material (*Ref 7, page 884*).^5^ They include risks that are not common, everyday matters, but which are known to occur on occasion. Similarly, a high risk of a slight injury can be material (*Ref 8, Para 27*).^6^

The scope of the duty of disclosure depends on the facts of each case. The Supreme Court of Canada expressly rejected the “professional disclosure standard,” which relies on what a reasonable doctor would disclose to a patient in similar circumstances to determine the scope of disclosure (*Ref 7, page 894*).^5^

In the context of elective procedures, Canadian courts have agreed that the *scope* of disclosure is greater (*Ref 9, page 179-180*).^7^ When deciding whether a risk is material, the elective nature of a procedure is a relevant factor that makes it much more likely for a risk to be assessed as a material risk (*Ref 9, page 180*).^7^

Failure to disclose a risk does not automatically lead to legal liability. It is not enough for a patient to allege that a physician has failed to disclose a material, special or unusual risk. The patient must also prove that it was the failure of the physician to provide information to the patient that caused the patient’s injuries. To determine whether such causation exists, the Canadian courts use the “modified objective test” (*Ref 7, page 897-900*).^5^ Under this test, causation exists if a reasonable person in the patient’s particular circumstances would have decided to forego the procedure had the patient been informed of the particular risk, which was not disclosed (*Ref 7*, *page 897-900*).^5^ In using this test, the court takes into account all objectively ascertainable circumstances of the patient that are relevant to the decision relating to the treatment (*Ref 10, Para 9*).^8^

### Informed Consent and STF Decisions

The focus of this review is to identify themes that arise from the STF Decisions and CMPA STF Data that relate to the informed consent process and discussions between physician and patient in the specific context of STF therapy.

#### Lack of comprehensive documentation of the consent discussion

One of the key trends in the STF Decisions and the CMPA STF Data is the role of thorough and detailed documentation of informed consent discussions in patient records when a Court or regulatory body assesses if informed consent has been obtained (Figure 2).

Many practitioners rely on consent forms to ensure that they have obtained informed consent. There is no question that a signed consent form is one component that will help a physician establish that informed consent has been obtained in the context of STF filler therapy.^9^

While the STF Decisions do rely on and reference the existence of a signed consent form to help determine that informed consent was obtained, the STF Decisions also indicate that the signed consent form is just one component of documenting informed consent.^10^ There is significant discussion in the STF Decisions about the importance of documentation in the patient record, apart from the consent form, to establish that a discussion has occurred between the physician and the patient, including the specific risks discussed.

For example, *Complainant v College of Physicians and Surgeons of British Columbia* suggests that regulatory bodies for physicians will be looking for physicians to clearly document a narrative outlining the discussions of the risk, benefits, and alternative treatments. In this decision, a patient complained to the College after developing complications from filler injections that she received over the course of a few treatments, including bruising, swelling, discoloration, indentation, and the development of lines in her face.^11^ The patient alleged, among other things, that she did not provide full and informed consent. The College determined that it was not possible to conclude that the patient underwent treatment without being advised of the medical and surgical risks of the procedure. However, the documentation of consent did not meet the College’s expectations, and regulatory criticism of the physician was warranted on that basis. The College stated that a consent form was not an adequate substitute for written documentation that informed consent had been obtained. In the particular circumstance of elective and optional procedures, the College directed that physicians need to clearly document a narrative to the effect that the risks, benefits, and alternative treatments have been discussed.

It is typical for patients to attend on more than one occasion and at regular intervals for STF therapy. A question arises as to how often the practitioner should be obtaining informed consent for repeat STF therapy. Although in the context of Botox, as opposed to filler, one decision provides some guidance on this issue. In *MBPM v REM*, the patient received Botox and other treatments from the physician in 2006 for migraines.^12^ After a 2-year absence, the patient returned for further Botox treatments for both therapeutic and aesthetic reasons, but the physician did not obtain a new written consent form at this time. The patient complained to the College about the physician’s use of therapeutic Botox for aesthetic reasons, alleging that it was done without consent. Based on the medical records, the College determined that the therapeutic Botox had been administered with “proper consent” in this case.

However, the College did comment that it would have been prudent for the physician to obtain new written consent for the patient when she returned from her 2-year absence, as well as being informative for the patient. The College stated its expectation that physicians should obtain patient consent for each procedure, a process which includes a discussion with the patient of the risks and benefits of the procedure and documentation of that discussion in the medical record.

Regulatory decision-makers indicate an expectation of a reasonably detailed documentation process for informed consent, consisting of both a signed consent form and documentation in the patient record that provides a narrative of the informed consent discussions with the patient each time that treatment is provided. In light of the STF Decisions, practitioners should consider implementing strategies to reflect in the record and on the consent form that the specific risks deemed to be material, special or unusual are documented in both the record and the consent form.

#### Lack of discussion of expected treatment outcomes

There are also intangible elements to patient care and communications that can have a direct impact on the physician-patient relationship, the quality of the communications between physician and patient and patient satisfaction. These aspects show respect and empathy with the patient’s own perception of what they wish to achieve, what will meet their expectations, a mood of cooperation, positive communication, and the time spent with the patient during their appointment impacts patient satisfaction.

Lack of discussion of expected treatment outcomes is also a theme that emerges from the STF Decisions and the CMPA STF Data as a factor that increases the medical-legal risk for physicians.^13^ These may include an untoward post-treatment course, downtime, facial symmetry, product migration, and no appreciable change, as well as the possible need and options for correction of unsatisfactory results.

#### Absence of discussion of complications associated with filler treatment

The major acute complication from STF treatment is vascular occlusion. If the occlusion is not one that is recognized or able to be reversed, the result is permanent tissue loss and scarring. The anatomical location of the sites of greatest frequency of vascular occlusion are well described; filler can traverse anastomotic arterial pathways causing obstruction at sites that are distant from the injection site.^14,15^ The patient who is about to undergo cosmetic therapy should be aware of the possibility of vascular occlusion, the options for management and the consequence of this adverse event if left untreated.

When injecting within the distribution of arteries that communicate with the ophthalmic artery, there is a risk of blindness. The incidence of blindness is very rare, with less than five medical-legal cases in Canada in the past 15 years (Table 3). This complication, however, is an irreversible, untreatable direct result of STF therapy. Consistent with the physician’s duty to disclose material, special, or unusual risks, the materiality of the devastating nature of this adverse event results in need to discuss this rare risk when injecting in areas that communicate with the ophthalmic artery. While the chance of infection developing from STF injection is expected to be low using best clinical practices, if infection were to develop, treatment would be required and part of the risk profile.^16^

Inflammatory nodules can develop many months or years after a filler has been placed in the skin. The incidence varies depending on the filler used, which tailors the injector’s consent discussion when reviewing the features of the product selected for each patient’s treatment.

The risks of infection, delayed inflammatory reactions, vascular occlusion, skin ulceration and blindness are low from a rate of incidence perspective. Ultimately, however, the courts will always engage in an examination of the patient’s circumstances and an assessment of what are material risks for that patient using the modified objective test described above to assess whether a reasonable person in the patient’s particular circumstances would have decided to forgo the procedure had the patient been informed of the particular risk which was not disclosed. While the risks of adverse events for STF therapy are low, they are material in terms of consequence for a cosmetic procedure, and jurisprudence accordingly suggests that they should be discussed with the typical patient in advance of therapy (Figure 1).

#### Delegation of Discussions

It is a practical reality of modern medicine that a patient attending a clinic for cosmetic procedures will be in the care of more than one health professional. In the context of multiple health professionals, it is common practice to have a nurse or other staff provide information about treatment to the patient. This raises the question of appropriate delegation of the duty of disclosure and obtaining informed consent.

From a general principles perspective, the law is clear that the physician or health professional who will be performing the treatment is the one who is ultimately responsible for ensuring that the patient is properly informed (*Ref 9, page 212*).^7^ The doctor who is performing the treatment will be held liable for incorrectly assuming that someone else, such as a nurse or junior colleague, has given the necessary information to the patient.

Despite bearing the ultimate responsibility, it is still acceptable for aspects of the duty of disclosure and obtaining informed consent to be delegated.^17,18^ Informed consent is a communication process, and whether or not the patient has been adequately informed and educated is the key issue when delegating the duty.^19^

#### Failure to Obtain Medical History, Understand Risk Factors and Lack of Thorough Patient Assessment

Cosmetic treatments carry the same obligations for understanding a patient’s purpose for attending therapy as with all medical or elective treatments. The practitioner should obtain the patient’s medical and surgical history, current medications, allergies and information about previous cosmetic or reconstructive facial procedures.^20-22^ This knowledge informs the practitioner about the patient. In the clinic setting, it is expected that there will be documentation of the patient’s goals, the practitioner’s clinical evaluation and assessment, the plan for management and the treatment that was carried out. Photographic documentation of the patient prior to treatment can provide a baseline for comparison for therapeutic results or changes after injection. Physicians open themselves up to increased medical risk by underestimating the importance of properly assessing a patient and suggesting treatment based on the assessment (Figure 2).^23^

## Conclusion

This study evaluated the medical legal cases related to the dermal filler in Canada, which closed with final determination between 2005 and 2019. The 90 cases exemplify the range of issues arising from STF injection that lead to civil litigation and complaints to regulatory authorities and those that also impact patient care and satisfaction.

The insights obtained from examining the CMPA STF Data, the peer expert evaluations and STF Decisions illustrate the importance of thorough and detailed documentation of informed consent discussions in patient records, extending beyond a signed consent form, and detailed discussions about expected treatment outcomes and potential complications of STF therapy tailored to each individual patient in order to obtain informed patient consent. Additionally, the clinic context gives rise to issues of effective processes for delegation of discussions about expected treatment outcomes and complications, as well as the importance of a physician’s understanding of each patient through a thorough patient assessment, including their medical history and risk factors.

When developing systems and procedures in their own clinical practice, practitioners should remain cognizant of these features that have historically given rise to medical-legal risk through litigation and regulatory proceedings.

## Supplemental Material

Online supplementary file 1 - Supplemental material for Soft Tissue Filler Therapy and Informed Consent: A Canadian Review Click here for additional data file. Supplemental material, Online supplementary file 1, for Soft Tissue Filler Therapy and Informed Consent: A Canadian Review by John P. Arlette, Andrea L. Froese and Jaspreet K. Singh in Journal of Cutaneous Medicine and Surgery
