# Supplementary material for: Soft Tissue Filler Therapy and Informed Consent: A Canadian Review
Source: J Cutan Med Surg. 2021 Jul 26;26(1):50–6. doi: 10.1177/12034754211032542 (PMC8750135; doi:10.1177/12034754211032542)
Supplement: Online supplementary file 1 - Supplemental material for Soft Tissue Filler Therapy and Informed Consent: A Canadian Review [file sj-pdf-1-cms-10.1177_12034754211032542.pdf]

Supplementary Material

SOFT TISSUE FILLER THERAPY AND INFORMED CONSENT–A CANADIAN REVIEW

John P. Arlette MD, FRCPC[1], Andrea L. Froese, BA, LLB[2], Jaspreet K. Singh, BHSc, MPP, JD[3]

[1] Clinical Professor, Department of Surgery, Cumming School of Medicine, University of Calgary, Alberta, Canada

[2] Partner, Bennett Jones LLP, Calgary, Alberta, Canada

[3] Associate, Bennett Jones LLP, Calgary, Alberta, Canada

Correspondence: John P. Arlette MD, FRCPC

Department of Surgery, Cumming School of Medicine, University of Calgary, Alberta, Canada

ORCID ID: 0000-0001-8582-9499 | Tel: (+1) 403-650-0515 | Email: john@johnarlette.com

Table S1. Summary of STF Decisions.

Case Name	Year	Venue	Issues	Case Summary
Sexton v Smith	2019	Litigation	Complications from filler injections and informed consent	The plaintiff received injections of Botox and Juvederm into her cheeks. Prior to this, the respondent physician set out the risks of Botox and filler procedures and provided a brochure about Juvederm. The plaintiff was able to ask any questions she had. The respondent did not warn the plaintiff about the risk of an MRSA infection, which the plaintiff subsequently developed. No lack of informed consent, as an MRSA infection is not a material risk of the procedures. Further, the court held that the plaintiff would have undergone the procedure even if apprised of the risks of infection.
RK v PRS	2018	College	Complications from filler injections and informed consent	Over ten months the applicant received four filler injections from the respondent for cosmetic treatments. The applicant developed pain, discomfort, and swelling to the right side of her face and mouth. Beyond the initial swelling with a hematoma, which eventually subsided, the Committee held that there was no objective finding of a negative outcome from the procedure. The patient completed a consent form for Botox and a consent form for dermal filler treatments. These consent forms were present in the medical record. The Board upheld the Committee's finding that the applicant had provided informed consent to the treatment.
Complainant v College of Physicians and Surgeons of British Columbia	2017	College	Complications from filler injections and informed consent	The patient first attended the physician's clinic to discuss non-surgical skin tightening to which the registrant recommended hyaluronic acid fillers to restore facial volume. The registrant completed the treatment, which resulted in lingering and significant swelling and bruising. Months later, the patient returned to the physician's clinic concerned about facial discolouration. A year after the initial treatment the patient complained that she now had more facial lines than ever before. The Committee's investigation of the physician concluded that while the patient understood the risks of the treatment and had consented, the physician's documentation of consent was inadequate.

Case Name	Year	Venue	Issues	Case Summary
Ontario (College of Physicians and Surgeons of Ontario) v Adams	2017	College	Complications from filler injections	Patient brought a complaint against the physician relating to injectable facial fillers treatment. Physician did not use the commercially available hyaluronic acid approved by Health Canada. He used hyaluronic acid as it was compounded by the pharmacy. Patient complained that the results quickly dissipated. Hollows had reappeared, along with the formation of a "ugly, hook or bow-shaped demarcation above the jaw line" and a "hard 1cm mobile lump" over the left mandible. The expert opinion stated that the use of compounded, rather than commercial, hyaluronic acid does not meet the standard of care. Further, the record was poorly charted, and contained no patient consent form or documentation of potential side effects of the procedure.
AD v MD	2014	College	Complications from filler injections and informed consent	Patient sought cosmetic treatment to correct the results of a Juvederm injection received at another clinic. Physician agreed to treat the applicant with hyaluronidase. The patient complained that the injection of hyaluronidase caused her entire face to melt. The respondent had discussed the risks of hyaluronidase injections, notably that it would dissolve hyaluronic acid in the face and could lead to dermal atrophy and asymmetry. Each time that treatment was provided, the respondent examined the applicant to ensure the requested treatment was appropriate, discussed the risks, and obtained a signed consent before proceeding with the treatment.
SK v CD	2014	College	Complications from filler injections	The patient received five plastic surgery procedures, each administered by the physician. Among these procedures, the patient received injections of Restylane under her eyes which resulted in a chemical burn and blurry vision.
AD v RSM	2014	College	Complications from filler injections	The patient received injections of hyaluronic acid in the form of Volumalift for concerns of loss of volume in the face. Later, the patient complained that the physician had injected her face with excessive amounts of filler. The Committee found that the physician used a very high volume of Volumalift and that there

Case Name	Year	Venue	Issues	Case Summary
				were more appropriate treatments for facial lines which would not have resulted in more lines over time. While the Board found that the patient had given informed consent, they noted that the physician displayed questionable ethics in representing hyaluronic acid as an appropriate remedy for facial lines and suggesting that it would improve the applicant's appearance.
MBPM v REM	2014	College	Informed Consent (Botox context)	The patient received Botox and other treatments from the physician in 2006. In 2007, the patient signed a general consent form and an ArteColl-specific consent. The patient received further Botox treatments between 2008 and 2010. After a two-year absence, the patient returned to the clinic in 2012 and received another Botox treatment. Despite an absence of two years, the physician did not obtain a new written consent from the applicant. The Committee held that the physician should have obtained new consents after the patient's absence from the clinic. Despite this, the Committee found that the patient did give consent.
College of Physician and Surgeons of British Columbia v Ezzati	2019	Litigation	Complications from filler injections and informed consent	Defendant had injected a woman, with dermal fillers. Defendant went through a consent form and the patient initialed each paragraph and signed at the bottom. Defendant showed a slideshow of different fillers, explaining the differences between them and the nuances of the procedure. Patient realized a bump had developed on her lip. The court did not address whether the bump was a complication of the Stylage, or poor administration by the Defendant. Defendant could not obtain informed consent because she was not licensed to perform medical procedures.
College of Physician and Surgeons of British Columbia v Ezzati	2021	Litigation	Complications from filler injections and informed consent	Defendant was in contempt of a court order and continued to hold herself out as a doctor and inject individuals with botulinum toxin, dermal fillers and other drugs.

**Case Name**	**Year**	**Venue**	**Issues**	**Case Summary**
Dowell v Millington	2016	Litigation	Complications of filler injection	The plaintiff brought a claim against the defendant owner of a spa for injuries sustained following an electrolysis procedure on her face. One week prior to the electrolysis, the plaintiff had received injections of Beautical 5, a dermal filler into her face. These injections occurred in areas where the subsequent electrolysis took place. Following the electrolysis, the plaintiff's face began to swell and became painful. The plaintiff attended a hospital three times before a doctor determined that the electrolysis had caused the adverse reaction after the injection of Beautical 5.
Martinez de Morales v LaFontaine-Rish Medical Group Ltd	2009	Litigation	Complications from filler injection	The plaintiff' attended for a lip augmentation procedure and claimed that her lower lip was injected with an unknown substance. The plaintiff developed a serious lower lip infection and was rushed to emergency care with a threat of lower lip necrosis. The plaintiff was awarded a default judgement.
Hoisington v Johnson & Johnson	2020	Litigation – Class Action	Complications from filler injections	The plaintiffs brought class action lawsuits in Ontario and British Columbia against multiple corporate defendants. The claim was based on the side effects of the cosmetic injectable lip filler Evolence. Initially, Evolence was marketed as a filler to repair deep facial lines. One of the defendants, began to advertise the product for use in lip augmentation. Following injection of Evolence, the plaintiffs alleged that they experienced permanent lip disfigurement. This disfigurement took the form of nodules, a change in lip colour, unusual swelling, chafing and chapping of the lips, and an increased sensitivity to cosmetics causing redness and inflammation. The court found that the defendants knew, or ought to have known that Evolence had not been tested for use as a lip augmentation filler. The defendants also knew, or ought to have known, that use of Evolence for purposes other than filling facial lines, in particular lip augmentation, would cause disfigurement, pain, irritation, and swelling.
Logan v Dermatech,	2011	Litigation – Class Action	Complications from filler injections	The plaintiffs brought a class action suit against the defendants for complications arising from the use of the defendants' product Dermalive. Dermalive is a product used to reduce wrinkles and

**Case Name**	**Year**	**Venue**	**Issues**	**Case Summary**
Intradermal Distribution Inc				other cosmetic characteristics associated with aging. Evidence before the court suggested that up to as many as 5.5% of all Dermalive users developed complications in the form of facial granulomas. The court certified the class action and allowed it to proceed.
R v Ashkani	2017	Litigation - Criminal	Complications from filler injections and informed consent	The accused held herself out as a registered nurse and injected Botox and collagen fillers into the faces of the complainants, each of whom suffered complications. When the accused performed the injections, she was not a nurse, nor was she under the direct supervision of a physician. The court found that all of the complainant's consents were vitiated by the accused's fraudulent claim that she was a registered nurse or under the supervision of a physician. The court held that the accused was guilty of assault with a weapon against each complainant.
